# Evaluation of the Effect of Improvers: Psyllium and Xanthan Gum in Bread Loaf with Partial Replacement of Quinoa Flour

**DOI:** 10.3390/foods14030418

**Published:** 2025-01-27

**Authors:** Laidy Quinte, Ingrid Valderrama, Ivan Best

**Affiliations:** 1 Carrera de Ingeniería Agroindustrial y Agronegocios, Facultad de Ingeniería, Universidad San Ignacio de Loyola, Lima 15024, Peru; laidy.quinte@usil.pe (L.Q.); ingrid.valderrama@usil.pe (I.V.); 2 Instituto de Ciencias de Los Alimentos y Nutrición, Universidad San Ignacio de Loyola (ICAN-USIL), Campus Pachacamac, Lima 15823, Peru

**Keywords:** bread loaf, quinoa flour, xanthan gum, Psyllium, breadmaking, acceptability, rheological properties

## Abstract

Wheat flour (WF) was replaced with quinoa flour (QF) at a 20% level in combination with improvers such as psyllium (PSY) and xanthan gum (XG). The flour quality, dough rheology, baking quality, and sensory evaluation of the bread loaves were analyzed, considering the addition of improvers as follows: PSY 0.5%, XG 0.5%, and PSY 0.25% + XG 0.25%. The best treatment to produce bread loaves was with the application of PSY 0.25% + XG 0.25%, where it had an optimal acceptability and no significant texture difference (*p* ≥ 0.05) compared with the control, with manageable dough, ideal viscosity, intermediate width/height ratio, and moderate baking stability, reflected in reduced baking losses. The techno-functional and nutritional properties of QF offer a viable alternative to WF.

## 1. Introduction

Wheat in Peru is a minor commercial crop, with production concentrated in the southern highlands, between 2800 and 3500 m above sea level. A significant quantity of wheat is produced by smallholder farmers and remains limited by difficult mountainous geography and rudimentary production practices. In 2023, Peru produced about 1.6 million metric tons of wheat flour, almost all of it from imported wheat. Similarly, the consumption and manufacture of wheat-derived products have remained at the same levels since 2021, with a slight increase in 2024, where 63 percent was destined for baking [1]. Replacing part of the wheat flour (WF) imports used in bakery products with flour from other starchy sources could be a viable solution [1,2]. Breads represent the most significant quantitative part (around 30%) of the total food consumed daily [3], located at the base of the food pyramid worldwide, with a consumption of 19.73 kg per capita in 2021 for the cities of Lima and Callao [4]. Quinoa (*Chenopodium quinoa* Willd.) is a grain originating from the Andean region with significant production in Peru, Ecuador [5], and Bolivia. It is highly adaptable and less demanding in terms of cultivation conditions [6]. There is also a growing trend in the consumption of healthy products, driven by current consumer demand. These products are expected to have a compound annual growth rate of 1.43% from 2019 to 2024 [7]. According to the World Health Organization (WHO), overweight and obesity in Peru are growing public health concerns. In recent years, there has been a significant increase in the number of adults affected. In 2022, obesity in urban areas rose to 27.8%, while in rural areas it increased to 16.2%. Regarding overweight, the rates were 38.5% and 32.8% respectively [8].

Wheat is the primary cereal used for bread making [9], with flour extracted through dry milling, a process that eliminates valuable nutrients and bioactive compounds [10]; therefore, it is essential to include flour based on cereals and pseudocereals to enhance nutritional value. Quinoa, for instance, is a rich source of high-quality proteins (14–18%) and amino acids [11]. Several studies show that the incorporation of quinoa flour (OF) into bread loaf formulations produces slight technological changes due to the dilution of gluten, as well as the inclusion of fiber and/or lipids at a 25% substitution [10], in addition to an increase in nutritional value and good acceptability. Gluten is responsible for the elasticity and strength of dough by forming linear structures through disulfide bonds [12]. An increase in the level of added quinoa causes the level of hardness to increase, probably because of the globulins and albumins, which are linked together through disulfide bridges and retain more water than the prolamins in wheat flour. A reduction in gluten in the dough matrix leads to low gas retention, which gives the formation of a harder dough [13].

The rheological and texture properties were not affected at a substitution of up to 20% [7] since it did not cause deformation of the mass [14]. A significant increase in the weight of the treatments was observed, as well as a reduction in the volume of the crumb because QF does not have gluten-forming protein. Wheat and wheat-quinoa bread loaves, with 20% substitution of QF, had a similar height; on the other hand, as the substitution percentage increased, the weight of the sample increased considerably [15]. The increase in the substitution levels of QF generates a gradual increase in the water absorption of the dough [16].

In recent years, the use of additives such as psyllium (PSY) and xanthan gum (XG) in breadmaking has garnered significant attention. Studies have investigated the effects of PSY husk on bread quality, revealing that increasing PSY levels enhance dough hydration capacity but reduces bread loaf volume [17]. In gluten-free bread formulations, the addition of PSY resulted in a softer, more resilient crumb, demonstrating its effectiveness as an anti-staling agent by significantly slowing the hardening process [18]. Research on the impact of XG (at doses ranging from 0.1% to 0.5%) on the rheological properties, dough development, gas formation, and baking performance of WF highlighted that a 0.2% dose produced bread of acceptable quality [19]. Further studies examining the effects of XG, guar, and Arabic gum (at doses of 0.5%, 1.0%, and 1.5%) on bread quality concluded that using 1.0% to 1.5% XG could yield superior bread compared with the control formulation [20]. Comparisons of PSY and XG on starch properties have shown that both hydrocolloids similarly increase the water absorption capacity of starch and induce rheological changes in gels when water is limited, with both also reducing gel hardness to the same extent [21]. Despite these findings, there are few studies on the effect on the baking and rheological quality of the integration of QF in the preparation of bread with the use of technological improvers (PSY and XG).

This study aimed to evaluate the acceptability through physical, chemical and sensory characteristics in the production of bread with partial replacement of QF considering the application of the improvers: PSY and XG (PSY 0.5%, XG 0.5%, and PSY 0.25% + XG 0.25%); to define the optimal acceptability having as control a bread with 100% wheat flour without improvers, quality parameters of the bread in terms of rheological properties of the dough and the characteristics of the final product. This is to strengthen the use of QF in the baking industry, resulting in increased income for the high Andean families producing Andean grains, as well as decreasing the usual use of WF in bakery products and reducing its importation.

## 2. Materials and Methods

### 2.1. Materials

Wheat flour (WF) was sourced from Molitalia SAC (Lima, Peru), derived from the Western Canadian red spring wheat variety, also known as CWRS. QF was produced from the white quinoa variety purchased from Nutrimix SAC (Lima, Peru), which collaborates directly with farmers in the Puno and Ayacucho regions in Peru. The improvers used in this study, XG and PSY, were procured from Ketolife SAC (Lima, Peru).

### 2.2. Methods

#### 2.2.1. Flour Quality Analysis

The analyses were conducted as follows: moisture [22]; ash (NTP 205.038) [23]; damaged starch (AACC Method 76-33.01) [24]; falling number (AACC Method 56–81.04) [25]; color using a digital colorimeter, model CR-400 (Konica Minolta, Tokyo, Japan) based on the 1976 definition by the International Commission on Illumination (CIE) [26], reported as L*, a*, and b*, representing the black-white, green-red, and blue-yellow ranges, respectively; protein (AOAC 979.09, Kjeldahl Method) [27].

#### 2.2.2. Rheological Analysis of the Dough

Rheological analyses were performed using Alveograph NG equipment (Chopin Technologies, Villeneuve-la-Garenne, France), following ICC Standard Method No. 121 [28]. The parameters recorded were tenacity (P), resistance to deformation, extensibility (L), maximum air volume that can form bubbles, P/L ratio, curve configuration, deformation energy or baking force (W), and area under the curve.

#### 2.2.3. Bread Making Process

Bread loaves were developed using the 10-10B straight simple dough method (AACC, 2000) [29]. All ingredients were calculated based on g/100 g of the flour mix, with wheat flour replaced by 20% QF (WF 80% and QF 20%). The concentration of QF was chosen based on the previous literature [2,10,30,31]. A control bread loaf was prepared using WF. PSY and XG were added to the formulation according to the experimental design at 0.5 g/100 g, resulting in PSY 0.5%, XG 0.5%, and PSY 0.25%/XG 0.25%. The ingredients, including WF, QF, PSY, XG, instant dry yeast (1.8%), salt (1.8%), oil (5.5%), margarine (9%), and sugar (6%), were combined, and water was added to achieve a water content of 54.6%. All ingredients were mixed in a Heavy-Duty pedestal mixer, model 5KSM7591XEWH, capacity 6.9 kg (KitchenAid, OH, USA) with ten working speeds, using the first two speeds to mix the ingredients and up to the fourth speed for kneading depending on the gluten development in the dough. Once kneading was completed, the dough was divided into 490 g portions. After resting for 30 min, the dough portions were placed in aluminum molds (198 mm × 110 mm × 65 mm) greased with butter. Fermentation was carried out for 90 min at 30 °C and 75% relative humidity. The molds were then placed in a convection oven, model BGS/13SC (Southbend, NC, USA), at 180 °C for 45 min. After baking, the bread loaf was cooled for 1 h at room temperature, packed in polypropylene bags, sealed, and stored at room temperature. All bread formulations were made in duplicate (two batches).

#### 2.2.4. Physicochemical Properties of Bread Loaf

The bread loaf volume was measured by the displacement of canary seed. The bread loaves were placed in a container of known volume, which was then filled with canary seed. The volume of seed displaced by the container represented the container’s volume.

The specific volume of the bread loaf was determined using an adaptation of the AACC 10-05.01 method (AACC, 2000) [32], with canary seed instead of rapeseed.

The specific volume of bread loaf was calculated using the following Equation (1):

(1) $$Specific\;volume\;(\frac{mL}{g})=\frac{volume\;of\;bread\;loaf\;(mL)}{weight\;of\;volumen\;(g)}$$

Baking loss was calculated as the difference between the initial dough weight and the bread loaf weight after cooling for 2 h, following the procedure described by Horstmann et al. [33].

The bread loaf weight (g) was measured using a precision balance, Serie FX-3000i (Brand A&D, Oxfordshire, UK). The bread loaf weight was calculated using the following Equation (2): The results were expressed as a percentage:

(2) $$Bread\;loaf\;weight\;\left(\%\right)=\frac{\left(dough\;weight-weight\;of\;cooled\;bread\;loaf\right)}{weight\;of\;cooled\;bread\;loaf}\times 100$$

The height and width of the bread loaves were measured at the center of the sample using a Vernier caliper.

The width/height ratio (*b*/*a*) of the slices of the bread loaves was calculated using the following Equation (3):

(3) $$b/a=\frac{\;width\;of\;the\;slice\;of\;bread\;loaf(cm)}{height\;of\;the\;slice\;of\;bread\;loaf\;\;(cm)}$$

The water activity (of bread loaf crumb) was measured at 20 °C using the AQUALAB 4TEV DUO equipment (Washington, DC, USA). The measurements were taken from four central slices of each bread loaf, according to Machado-Alencari et al. [34]. All measurements were performed in triplicate.

Crumb pH was determined using the AACC 02-52.01 methodology (AACC, 2000) [29] with a Multi 3620 IDS potentiometer (Weilheim, Germany). The readings were taken in triplicate.

#### 2.2.5. Crust and Crumb Color of Bread Loaf

Color measurements (CIE, L*, a*, b*) were obtained using a digital colorimeter, model CR-400, (Konica Minolta, Tokyo, Japan) equipped with a standard D65 illuminant, previously calibrated with a blank plate. The results were the averages of three measurements of L* (brightness; 0: black, 100: white), a* (red–green), and b* (yellow–blue), in addition to chromaticity (C*) and hue angle (h°). The crumb and crust color were measured at three preselected points for each treatment [35].

#### 2.2.6. Proximate Analysis of Bread Loaf

The analyses were performed as follows: moisture (AOAC Official Method 920.87) [27]; ash (AOAC 935.39 (B)) [27]; fat (AOAC Official Method 935.39 (D)) [27]; protein (AOAC Official Method 950.36) [27]; crude fiber (NTP 205.003 1980. Rev. 2011) [36]; carbohydrate [37]; total energy [37]; %kcal [37].

#### 2.2.7. Sensory Analysis of Bread Loaf

The bread loaves were evaluated by a panel of 20 untrained panelists aged between 20 and 55 years. They received instructions and were given samples of bread loaf accompanied by a glass of water, then proceeded to answer the provided questionnaire. The following variables were evaluated: appearance, color, flavor, texture, and acceptability, using a 5-point hedonic scale (1 = dislikes a lot; 2 = dislikes a little; 3 = neither likes nor dislikes; 4 = likes a little; 5 = likes a lot). The panelists were instructed to rinse their mouths between the evaluations.

#### 2.2.8. Experimental Design

In the first stage, flour quality was evaluated, with the control being the treatment based on 100% WF, while the other treatments were replaced with 20% QF. The application of 0.5% improvers was estimated as treatments as follows: PSY 0.5%, XG 0.5%, and PSY 0.25% + XG 0.25%, which included evaluation of physicochemical analysis, colorimetry, and rheological analysis. In the second stage, the preparation of bread loaves for all treatments was developed, where physical, physicochemical, and colorimetric evaluation of the bread loaves were conducted. In the third stage, sensory evaluations of the treatments were performed to choose the best treatment based on the control.

It is applied research with a true/pure experimental design:

For the first phase

$$\mathrm{ST}:\;X\;O_{1}$$

$$\mathrm{SC}:\;O_{2}$$

where:

X: Application of percentages of improvers (PSY and XG).
ST: Substitution of 20% WF with QF (20% QF; 80% WF).
SC: Standard/control.
O_2_: Measurement of moisture (%M), ash (%A), protein (%), falling number (FN), and damaged starch (UCD units) in the standard.
Colorimetry measurement (CIE *L*, a*, b*) in the standard.
Measurement of strength (W), tenacity (P), extensibility (L), and P/L ratio in the standard.
O_1_: Measurement of moisture (%M), ash (%A), protein (%), falling number (FN), and damaged starch (UCD units) in the experimental treatments.
Colorimetry measurement (CIE L, a*, b*) in the experimental treatments.
Measurement of strength (W), dough tenacity (P), dough extensibility (L), and P/L ratio in the experimental treatments.

For the second phase

ST: X O_1_

SC: O_2_

where:

X: Application of percentages of improvers (PSY and XG).
ST: Substitution of 20% WF with QF (20% QF; 80% WF).
SC: Standard/control.
O_2_: Measurement of specific volume (sp. vol.), *b/a* ratio, weight loss (%), water activity (aw) and pH of standard bread loaf moisture (%M), ash (%A), fat (%), protein (%), crude fiber (%) and energy (kcal/100 g) of standard bread loaf.
Colorimetry measurement of the crust and crumb (CIE *L*, a*, b*) of standard bread loaf.
O_1_: Measurement of specific volume (sp. vol.), *b/a* ratio, weight loss (%), water activity (aw), and pH of the bread loaf of experimental treatments.
Moisture (%M), ash (%A), fat (%), protein (%), crude fiber (%), and energy (kcal/100g) of the bread loaf of experimental treatments.
Colorimetry measurement of the crust and crumb (CIE *L*, a*, b*) of the bread loaf of experimental treatments.

In the third phase

ST: X O_1_

SC: O_2_

where:

X: Application of percentages of improvers (PSY y XG).
ST: Substitution of 20% WF by QF (20% QF; /80% WF).
SC: Standard/control.
O_2_: Sensory measurement of standard bread loaf.
O_2_: Sensory measurement of the bread loaf of the experimental treatments.

#### 2.2.9. Statistical Analysis

For phases I, II, and III, statistical treatments were performed using analysis of variance (ANOVA), or the Kruskal–Wallis test was used as appropriate. For variables with a normal distribution, the Tukey test was then applied to compare the means at a significance level of *p* < 0.05. This analysis was performed using Minitab 19 statistical software (State College, PA, USA) and IBM SPSS statistical software (test version 23.0, IBM, New York, NY, USA). These data represented the mean ± standard deviation.

## 3. Results and Discussion

### 3.1. Phase I: Analysis of the Flour Quality

#### 3.1.1. Physicochemical Characteristics of the Flour

Table 1 shows the physicochemical analysis of the raw materials, WF and QF, as well as the six treatments. The treatments evaluated in this study presented moisture, ash, protein, and damaged starch content that varied from 7.32% to 13.85%, 0.59% to 2.18%, 13.17% to 14.15%, and 5.13% to 8.33%, respectively.

WF presented a moisture content ranging from 13.70 to 13.75%, complying with the Peruvian technical standard (NTP) 205.064 (2015), which allows a maximum moisture content of 15% [38], and values close to 13.67% reported by Best et al. [39]. QF showed a moisture content ranging from 7.32 to 7.38%, lower than the values reported in some studies, 13.71% [40], and 13.20% [41], but higher than that found by Mamani et al. (6.03%) [42]. This moisture content does not exceed the maximum value of 13.50% established by NTP 011.451 (2018) [43]. WF had significantly higher moisture compared with the QF and composite flour treatments.

In general, Li et al. (2013) found that moisture levels of 14% or lower allow WFs to remain stable at room temperature and prevent the growth of microorganisms [44]. The low moisture observed in QF and WF was a good indicator of their potential for longer shelf life [45]. The lower the initial moisture content of a product to be stored, the better the stability during storage, as reported by Awoyale et al. [46].

According to Table 1, protein, ash, and damaged starch characteristics were significantly different (*p* < 0.05) based on the type of base flour. Regarding the ash content, WF presented a value ranging from 0.58 to 0.62%, which corresponds to that described in NTP 205.064 (2015) [38], a significantly lower value compared with the other treatments.

The treatments with composite flours showed varying values, ranging from 0.71 to 0.85%, which are lower than those reported by Coţovanu et al. (2023) in the range of 0.85–0.95% and with a value of 0.65% for wheat flour [9]. QF presented ash values aligning with the NTP 011.451 (2018) [43], a value close to 2.0% reported by Franco et al. (2021) [7] and higher than 1.91% reported by El-Said et al. [41].

The ash content is associated with the presence of bran particles, the outer layer of the wheat grain, in the flour. During milling, bran must be separated from the germ of the endosperm, as bran is responsible for the dark coloration [47]. Additionally, the ash content is related to the amount of minerals present in the grain and directly affects flour color [48]. It is worth noting that ash content and the level of extraction during the milling process are directly proportional. This relationship facilitates the classification of flours according to the degree of extraction: the lower the separation of the pericarp, the higher the ash value, which in turn increases flour extraction [49].

Regarding the protein content, WF showed values ranging from 13.18 to 13.23%, and QF, however, presented protein ranging from 14.03 to 14.15%. Flours with 20% QF substitution had protein values ranging from 13.20 to 13.85%.

Table 1 shows that the addition of XG increased the moisture value in treatments to respect the sample without improver (WF 80%–QF 20%) [50]. Similarly, the addition of PSY increased moisture and decreased ash [40]. In general, protein levels were significantly lower compared with QF but higher than WF.

The degree of starch damage is defined as the percentage of starch that is subject to enzymatic hydrolysis [51,52]. The damaged starch content of WF ranged from 5.37 to 5.42%, which is lower than the 5.73% reported by El-Said et al. (2021) [41]. The values of damaged starch also depend on the characteristics of the wheat and the milling conditions [53].

Regarding these findings, Jukic et al. (2019) found a range of damaged starch from 4 to 10% for baking flour [53]; however, QF had a significantly higher damaged starch content compared with the other treatments, ranging from 8.22 to 8.33%, lower than the value reported by Srichuwong et al. (2017) at 10.6% [54]. It is worth mentioning that the level of damaged starch and flour particle size are inversely proportional [51,52]. Higher water absorption may be associated with the influence of starch damage and the effect of flour particle size, as smaller particles have a larger total surface area [53]. Damaged starch, according to UCD units, is in an established range of 12 to 28 UCD units for WFs [55]. It is important to note that the flour sample comes from medium-hardness wheat, so the damaged starch should be low, as its value increases with the hardness of the grain. The value obtained is close to the optimum level of damaged starch [55].

#### 3.1.2. Flour Color Characteristics

Color is an important quality characteristic that strongly influences the consumer acceptability of flour and its by-products [44]. In this study, the color of the treatments shown in Table 2 was analyzed. The lightness (L*) value in WF was higher than that of QF, with values ranging from 89.99 to 90.08 and 85.28 to 85.57, respectively. Lightness in foods varies from L* = 0 (black) to L* = 100 (white), indicating that WF tends toward a white color, as evidenced in Table 2. As reported by Dussán-Sarria et al. (2019), QF presents L* values of 84.87, a* of 1.33, and b* of 14.87 [56], similar to the present investigation, except for the higher a* value. The L* values of the treatments with composite flours indicate higher lightness than quinoa close to the standard. Treatments with improvers (XG, PSY) did not have negative effects or significant differences in color at 0.5%, maintaining values close to the WF80-QF20 treatment, which does not contain improvers. It was also observed that the L* averages for the two treatments were significantly different from the other treatments (*p* < 0.05). Our results show that all treatments had L* averages significantly lower than the 100% WF treatment and higher than the 100% QF (*p* < 0.05).

The color coordinate a*, which ranges from −a* (green) to +a* (red), presented values ranged from −1.00 to −0.97 in wheat flour, while QF had 0.34 to 0.43. This value was also higher for the composite flour treatments due to the substitution of 20% WF with QF. According to Rojas-Garbanzo et al. (2016), high positive values of the a* coordinate indicate a higher content of carotenoids, pigments found in quinoa flour [57]. The treatments containing XG showed negative values, while those with PSY had positive values, both increasing as the proportion of the corresponding improver increased.

Regarding the values of the b* coordinate, which range from −b* (blue) to +b* (yellow), WF presented a lower value than QF; values ranged from 11.25 to 11.42 and 15.34 to 15.89, respectively, indicating a clear tendency toward yellow coloration [56]. A higher b* value is associated with the content of xanthophylls in the flour [47]. It is also observed that the b* averages for the two treatments are significantly different from the other treatments (*p* < 0.05). Our results show that all treatments had b* averages significantly higher than the average for the 100% wheat flour treatment and lower than the 100% quinoa flour treatment (*p* < 0.05).

#### 3.1.3. Characteristics of Dough Rheology Analysis

The falling number is negatively correlated with the activity of α-amylase and damaged starch in the WF [58]. In our study, the FN of the WF showed values ranging from 397.00 to 410.00 s, higher than the values reported in the literature, 347.80 s [9] and 312.00 s [41]. Calaveras (2004) indicates that the FN of medium-strength or bakery flour ranges from 325.00 to 400.00 s, a value close to the maximum obtained in WF. According to Coţovanu et al. (2023), the particle size of QF in samples with partial replacement of 20% affects the FN values, where an increasing trend of FN can be observed as the particle sizes are reduced [9]. Regarding QF, the FN values were very high, exceeding the typical values of wheat flour (more than 1000 s), similar to that reported by Tamba-Berehoiu et al. (2019). They indicate that QF quickly forms gels of greater consistency and stability than WF [31]. The composite flour without the addition of improvers showed high values compared with the control and the sample formulations containing XG and XG-PSY; however, the treatment containing PSY presents a higher value ranging from 423.00 to 447.00 s compared with the other treatments with improvers. This may be due to the capacity of the starch granule and the PSY fiber to retain water and swell freely, transforming into a mass with increasing viscosity, which leads to a decrease in α-amylase activity [59]. Variations in the FN for composite flours are related to quinoa starch. The specific surface of quinoa starch is greater than that of wheat, making it more sensitive to α-amylase hydrolysis. Additionally, quinoa has lower amylase activity, which increases gas production and, consequently, the bread loaf volume [9]. The FN is significant during dough handling; an FN below 200 s indicates excess α-amylase in the starch, leading to poor flour quality, dark crumb [60], and stickier dough [48]. Conversely, values above 300 s indicate low enzymatic activity and good quality wheat or flour, resulting in a less adhesive, elastic, cohesive, and resistant flour/water mixture, showing starch breakdown due to α-amylase activity [61]. The higher the number, the higher the viscosity and the lower the α-amylase activity. FN values above 250 s are generally accepted for bread loaf making [62].

Substituting WF is a major technological challenge since gluten is essential for forming the dough structure, responsible for the elasticity and extensibility properties needed for good quality bread loaf [63]. These properties of wheat doughs are particularly important in the process of making baked goods. The results of the alveographic analysis of wheat flours and treatments with QF are presented in Table 3. The addition of 20% QF and 0.5% improvers (PSY and XG) significantly affected (*p* ≤ 0.05) the alveographic properties of the dough. The tenacity (P) of the dough and the configuration ratio of the alveograph curve (P/L) increased, while the extensibility (L) of the dough and the baking resistance (W) decreased as the improvers were added to the flour mixture. Our results indicate a weakening effect when the improvers are added to the substituted dough, with the W value being lower than the control. PSY and XG increase their value as the added proportion increases, with less significant values for XG. The combination of both improvers caused the W value to remain constant at 0.5% addition. The tenacity of a dough (P) refers to its resistance to breaking and is associated with the glutenin content. It also measures the dough’s elasticity, which is necessary for retaining carbon dioxide (CO_2_) and forming the spongy structure generated by the denaturation of the glutelins during kneading. It is crucial for bakery dough to be strong to prevent gas from escaping easily [64]. The P value is related to the stiffness and/or tenacity of the dough, which increases with the damaged starch content, as more damaged starch equates to greater water absorption capacity of the flour [64]. Consequently, increasing the proportion of flour with high-damage starch in a mixture with low-damage starch flour will result in a linear increase in P and a reduction in dough in L [65]. This situation is reflected in the results, where it is evident that P increased as the WF content decreased due to the dilution of gluten in the dough, which aligns with another research [31,66,67]. The bread formulation with only QF reported low values ranging from 129.00 to 143.00 mm, followed by those containing PSY-XG, PSY, and XG improvers, all higher than the control treatment to 84.00 mm. The addition of improvers increased the tenacity of the treatments, with significantly similar values, due to the presence of fiber. The interactions between the polysaccharides of the fiber and the wheat proteins could generate increases in P [13], where PSY is a source of soluble fiber (70%), insoluble fiber (17%) [58], and XG contains soluble fiber at 77.8% [68].

The extensibility (L) analysis showed that the preparation with QF had significantly lower averages than the formulation with 100% WF (*p* < 0.05). The results indicated that the addition of QF decreases L [65]. This can be explained by the increased competition for available water, as shown in previous works [31]. The dilution of gluten and the decrease in its quality and quantity is due to the addition of QF, which contains proteins such as globulins and albumins that retain more water than wheat protein. This indicates that the gluten network of the composite dough was diluted, and the activity of α-amylase decreased, affecting the fermentation process [13]. Regarding the addition of improvers, it was found that the L values of the formulations with composite flours without improvers and the addition of PSY at 0.5% are significantly similar. These values are high compared with the treatments with XG and the one containing combined improvers (PSY/XG). The lowest value was obtained by the treatment with XG at 0.5%, indicating that bread formulations containing PSY have higher L values than those with XG. Generally, the decrease in L when adding improvers could be due to the high water absorption of fiber in doughs [69]. Additionally, PSY contains arabinoxylans and many hydroxyl groups, which capture water that would otherwise bind to gliadin and glutenin for gluten network development [67]. Consequently, the formed gluten is weak, leading to a significant reduction in L. However, this did not negatively affect the W value (area under the curve), so the rheological characteristics of WF dough remained unaffected [70,71]. Furthermore, XG increases the dough’s resistance to alveograph extension (P) while decreasing L [72].

The addition of QF significantly decreased the strength (W) (mechanical work of the alveograph) of the dough, influenced by the P/L ratio of the control. As shown in Table 3, although the mechanical work of the WF was higher, ranging from 274.00 to 287.00 10^−4^ J/g, due to small initial extensibility, the ability to maintain or increase the mechanical work of the dough by adding QFs was extremely low, ranging from 173.00 to 252.00 10^−4^ J/g with the addition of improvers. Additionally, the alveographic analysis was conducted under constant hydration conditions; therefore, the addition of quinoa flours can influence the formation of more consistent doughs from a rheological perspective [31]. The results obtained align with those mentioned by other authors [9].

The balance between P and L, also known as the configuration ratio (P/L), is used to predict the baking capacity of wheat flour [55]. In Table 3, it can be observed that the equilibrium value of the treatment with the addition of QF is higher compared with the control, with values ranging from 2.40 to 2.98 and 0.74 to 0.87, respectively. It is lower than that of the composite flours with the addition of improvers. The magnitude of the increase in the P/L value is influenced by the type of improver added, with high, intermediate, and low values presented for the treatments containing XG, XG /PSY, and PSY, respectively. The increase in the P/L ratio is attributable to strong interactions of XG with flour proteins [72]. Additionally, the increasing P/L value is directly proportional to the increase in the dosage of the improvers, influenced by the tenacity and extensibility resulting from the type of flour. High tenacity combined with decreasing extensibility results in high P/L values, as seen in the treatments with partial substitution of QF.

### 3.2. Phase II: Analysis of the Quality of the Bread Loaf

#### 3.2.1. Physical Characteristics of Bread Loaf

The physical characteristics of the bread loaf are shown in Table 4, where the *b/a*, specific volume, weight loss, aw, and pH of the 100% WF and the composite flours without improver were evaluated, as well as the addition of improvers separately and the synergy effect when combined.

The width/height ratio (*b/a*) of the central bread loaf is related to the stability of the dough during baking; high values indicate lower stability [73]. In our study, no significant differences were observed in the *b/a* ratio between the different treatments. These values are higher compared with other studies [73,74].

However, we found that the specific volume in the bread loaf from the control treatment with 100% WF (2.52 cm^3^/g) was higher than that reported in the literature (2.45 cm^3^/g) [13], which is close to the treatment of composite flour with the addition of 0.5% PSY (2.39 cm^3^/g). The results differ from the treatment without the addition of improvers, which shows a low specific volume of 2.13 cm^3^/g. The specific volume of the baked bread loaf is affected by the addition of QF, as the substitution of WF with no glutinous flour reduces the gluten content and, therefore, the volume of the bread loaf. Furthermore, the proteins (globulins and albumins) in QF capture more water than wheat, indicating that the gluten network of the composite dough was diluted, reducing the activity of α-amylase that influences the fermentation process [75]. Regarding the composite flours with the addition of improvers, values significantly similar to the control are observed, with a slight decreasing effect, ranging from 2.29 to 2.39 cm^3^/g. According to Franco and Gómez (2022), the incorporation of PSY does not affect the specific volume of the bread loaf or the weight loss during baking [38], contrary to what was reported by Shittu et al. (2009), who found that increasing the level of PSY decreases the specific volume of the bread loaf, an effect regulated by increasing the water level [76]. Zannini et al. (2014) indicate that the addition of XG had no significant effect on the specific volume of bread loaf, attributable to the strong gluten-hydrocolloid relationship that prevented the extension of the dough [77].

The weight loss of the bread loaf is due to the evaporation of water during baking and cooling [30]. The treatment of composite flour with XG at 0.5% showed a higher weight loss than the control. Additionally, the treatment with both improvers (PSY and XG) at 0.5% showed a lower weight loss compared with all treatments. The value of the cooking loss should not exceed 9%, as it would indicate overcooking [30].

The bread loaves produced do not present significant differences in water activity (aw), an important indicator of the microbiological stability of the bread loaves. The aw delays microbial deterioration in foods, potentially leading to prolonged shelf life of the product [78]. Therefore, the lower the moisture content, the greater the durability [79]. It is also an indicator of sensory properties, such as aroma, flavor, and texture [80]. The aw values obtained are in the range of 0.95 to 0.97, indicating that the addition of improvers did not affect the aw values. Our results are similar to those reported by Wang et al. (2015) [78] and contrary to Rosell et al. (2001), who reported an increase in aw because the addition of improvers increases the water retention capacity [81].

The pH values range from 4.90 to 5.41 for the treatments of composite flour bread loaves and 5.33 to 5.40 for the control bread loaf based on 100% WF. These values are lower than the range reported by De la Cruz (2009), which ranges from 5.23 to 5.61 for bread loaves with a partial substitution of up to 20% QF [82]. It is worth mentioning that the pH value is related to the acids produced during fermentation [6].

#### 3.2.2. Physicochemical Characteristics of Bread Loaf

Table 5 shows that the moisture percentage ranges from 32.02% to 34.16%. This confirms the downward trend toward greater substitution with QF in bread loaves. From Table 5, it is evident that the substituted bread loaves showed a reduction in moisture content, suggesting that quinoa starch granules have a greater water absorption capacity than those of WF.

It has been determined that the ash and fiber levels of all treatments with the addition of improvers (PSY and XG) have increased significantly, while their fat and carbohydrate levels are lower compared with the treatment with 100% WF. This shows the influence of QF within the dough as it contributes to nutritional enrichment [2,3,4].

Quinoa has a higher protein content compared with other Andean grains cereals [83]; this causes the nutritional value of bread loaves with partial substitution of quinoa flour to improve significantly compared with conventional bread made with 100% wheat flour.

#### 3.2.3. Characteristics of the Color of the Crust and Crumb of Bread Loaf

Table 6 shows the color values expressed as L*, a*, b*, evaluated in the substituted bread loaf formulations, as well as the effect of using the improvers PSY and XG. The color (or appearance) attributes of the crust and crumb are composite properties of baked products that are determined in part by the degree of the Maillard reaction [75].

**Figure 1:**
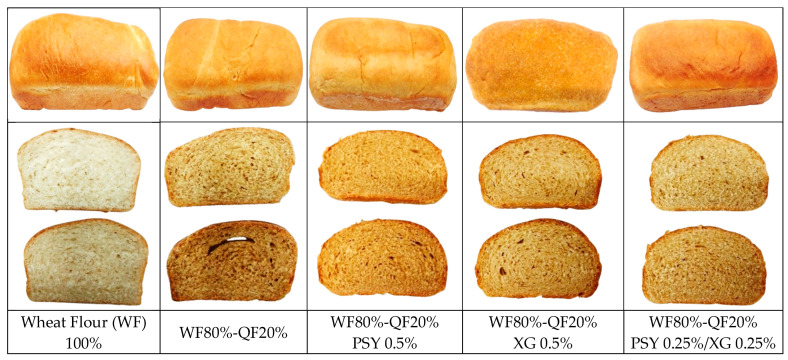
Bread loaf of WF and treatments. WF: wheat flour; QF: quinoa flour; PSY: psyllium; XG: xanthan gum.

In the outer crust of the bread loaves, the value of L*, which indicates lightness and varies from L* = 0 (black) to L* = 100 (white), shows that formulations with QF with partial substitution and the addition of improvers have a statistically lower L* level, ranging between 45.82 and 50.44, compared with the control bread loaf value ranged from 52.43 to 53.67. They also presented greater lightness compared with bread loaf without improvers. Rosell et al. (2009) mention that there is a reduction in L* of the crumb as wheat is replaced with other Andean grain flours (quinoa, tarwi, kañiwa, and kiwicha). This is more evident when comparing the standard WF 100% bread with the treatments containing QF; however, the XG improver is closer to the standard (Figure 1) [84].

The a* values, which range from −a* (green) to +a* (red), in the crust of all bread treatments indicate a greater tendency toward red (+), with no significant differences among the bread. In the measurement of the center of the crumb, all treatments have lower a* values than the crust; however, the bread loaf with 0.5% PSY obtained a higher value compared with the other bread loaves.

The b* values ranged from −b* (blue) to +b* (yellow). All treatments presented positive b* values. Regarding the crust of the bread loaf samples, the b* value showed a greater tendency toward yellow in the 100% WF control bread loaf, while the bread loaves with partial quinoa substitution had a slight decrease in yellow intensity.

By adding QF and using improvers, there was an increase in the b* value in the bread loaf crumbs, which was slightly higher than the control bread loaf, being more noticeable in the bread loaves with 0.5% PSY QF. According to Chisenga et al. (2020), the development of a yellowish color (higher b*) could be attributable to the nonenzymatic reaction between the reducing sugar and proteins present in the flour [85].

### 3.3. Phase III: Sensory Analysis of Bread Loaf

Sensory Evaluation of Bread Loaf

**Figure 2:**
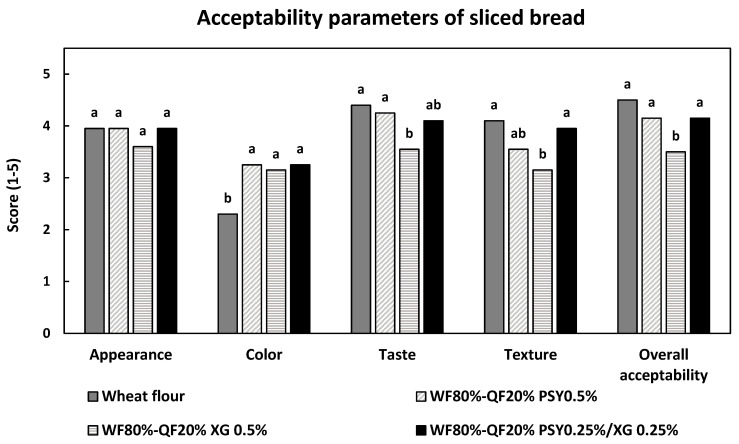
Sensory analysis of bread loaf. WF: wheat flour; QF: quinoa flour; PSY: psyllium; XG: xanthan gum. The numbers “1” to “5” indicated the preference (1: dislikes a lot; 2: dislikes a little; 3: neither likes nor dislikes; 4: likes a little; 5: likes a lot). Values with different letters (a, b) are significantly different (One-way ANOVA with Tukey’s multiple comparison test, *p* < 0.05). All the readings were taken in duplicates.

Based on the sensory evaluation of the bread loaf, it was established that, with a confidence level of 95%, there are a few differences between treatments. The sensory profile of the treatments for the five characteristics showed that the highest acceptability similar to the control was obtained by the treatments with the addition of PSY 0.5% and PSY 0.25%/XG 0.25% (Figure 2)**.**

However, the treatment with the addition of PSY 0.5% and the treatment with 0.25% PSY/0.25% XG achieved the taste characteristic closest to the control. In terms of appearance, there are no significant differences between all the evaluated treatments, and with respect to color, a higher score is observed in the treatments with the addition of QF, compared with the control, since the addition of QF caused the bread loaves to have a more attractive color, because of the Maillard reaction in baked products.

## 4. Conclusions

This study revealed that with a 20% substitution of quinoa flour and the addition of improvers, it is possible to produce a bread loaf with sensory qualities similar to the control. The incorporation of improvers (PSY and XG) resulted in an improvement in the quality of the bread loaf, as bread loaves with a greater volume and a specific volume close to the control were obtained, with PSY 0.5% obtaining the highest value, thus reducing the impact of the addition of quinoa flour that significantly affects the volume of the bread loaf.

The option of partial substitution highlights the nutritional benefits and contributions of quinoa flour and the quality provided by the improvers, making it an alternative substitute for conventional bread loaf. The addition of PSY 0.25%/XG 0.25% improvers demonstrated a specific volume close to the control, as a falling number within the parameter was reached, with manageable dough and ideal viscosity. The intermediate *b/a* ratio provides moderate baking stability, reflected in lower cooking loss, resulting in a soft and porous texture for the bread loaf.

Future studies should focus on investigating substitutions between different flour compositions based on Andean grains or alternative ingredients, thus further optimizing the formulation of bread. This will undoubtedly deepen our knowledge and broaden the scope of possibilities for creating good quality bakery products.

The scientific value of the paper lies in its contribution to the development of good quality bread, with a focus on the effect of improvers in combination and individually in the mixture of substituted flours and the analysis of dough behavior and various aspects of the bread-making process. It provides useful information both for the food industry and for future research to improve products with partial substitution. Similarly, focus should be placed on investigating the shelf life as well as the market potential of processed bread under an investigation of its acceptability.

## Figures and Tables

**Table 1:** Physicochemical properties of wheat flour (WF), white quinoa flour (QF), and treatments.

Parameter/Flour Type	Moisture (%)	Ash (%)	Protein (%)	Damaged Starch (%)
Wheat flour (WF)	${13.720\pm 0.026}^{\;\;a}$	${0.597\pm 0.006}^{\;c}$	${13.203\pm 0.025}^{\;c}$	${5.395\pm 0.035}^{\;c}$
White quinoa flour (QF)	${7.347\pm 0.031}^{\;d}$	${2.123\pm 0.067}^{\;a}$	${14.100\pm 0.062}^{\;a}$	${8.310\pm 0.028}^{\;a}$
WF 80%–QF 20%	${12.577\pm 0.159}^{\;c}$	${0.800\pm 0.036}^{\;b}$	${13.753\pm 0.087}^{\;b}$	${5.785\pm 0.021}^{\;b}$
WF 80%–QF 20% PSY 0.5%	${12.947\pm 0.150}^{\;b}$	${0.790\pm 0.053}^{\;b}$	${13.267\pm 0.055}^{\;c}$	${5.145\pm 0.02}^{\;d}$
WF 80%–QF 20% XG 0.5%	${12.907\pm 0.095}^{\;b}$	${0.793\pm 0.015}^{\;b}$	${13.253\pm 0.045}^{\;c}$	${5.230\pm 0.057}^{\;c,d}$
WF 80%–QF 20% PSY 0.25%/XG 0.25%	${12.893\pm 0.035}^{\;b}$	${0.773\pm 0.071}^{\;b}$	${13.207\pm 0.040}^{\;c}$	${5.345\pm 0.106}^{\;c,\;d}$

WF: wheat flour; QF: quinoa flour; PSY: psyllium; XG: xanthan gum. Values in the same column with different letters (a–d) are significantly different (One-way ANOVA with Tukey’s multiple comparison test, *p* < 0.05). Data are the mean ± standard deviation (n = 3).

**Table 2:** Chromatic properties of wheat flour (WF), white quinoa flour (QF), and treatments.

Parameter/Flour Type	L* (Lightness)	a* (Red/Green)	b* (Blue/Yellow)
Wheat flour (WF)	${90.033\pm 0.045}^{\;a}$	${-0.980\pm 0.017}^{\;f}$	${11.347\pm 0.087}^{\;c}$
White quinoa flour (QF)	${85.403\pm 0.150}^{\;f}$	${0.380\pm 0.046}^{\;a}$	${15.557\pm 0.293}^{\;a}$
WF 80%–QF 20%	${85.940\pm 0.036}^{\;e}$	${0.233\pm 0.015}^{\;b}$	${14.110\pm 0.209}^{\;b}$
WF 80%–QF 20% PSY 0.5%	${86.140\pm 0.036}^{\;d}$	${0.097\pm 0.012}^{\;c}$	${14.283\pm 0.116}^{\;b}$
WF 80%–QF 20% XG 0.5%	${86.523\pm 0.047}^{\;c}$	${-0.013\pm 0.006}^{\;d}$	${14.183\pm 0.102}^{\;b}$
WF 80%–QF 20% PSY 0.25%/XG 0.25%	${86.847\pm 0.021}^{\;b}$	${-0.107\pm 0.015}^{\;c}$	${13.970\pm 0.072}^{\;b}$

WF: wheat flour; QF: quinoa flour; PSY: psyllium; XG: xanthan gum. Values in the same column with different letters (a–f) are significantly different (One-way ANOVA with Tukey’s multiple comparison test, *p* < 0.05). Data are the mean ± standard deviation (n = 3).

**Table 3:** Falling Number (FN) and Alveograph analysis for wheat flour (WF), white quinoa flour (QF), and bread formulations.

Parameter/Flour Type	Falling Number (FN)	Tenacity P (mm)	Extensibility L (mm)	Mechanical Work, W (10^−4^ J/g)	P/L Ratio
Wheat flour (WF)	${404.000\pm 6.557}^{\;c,d}$	${84.000\pm 0.000}^{\;d}$	${105.000\pm 8.000}^{\;a}$	${281.333\pm 6.658}^{\;a}$	${0.803\pm 0.065}^{\;e}$
White quinoa flour (QF)	>1000	ud	ud	ud	ud
WF 80%–QF 20%	${513.000\pm 4.359}^{\;a}$	${137.333\pm 7.371}^{\;c}$	${46.000\pm 3.606}^{\;b}$	${187.333\pm 12.897}^{\;d}$	${3.003\pm 0.386}^{\;d}$
WF 80%–QF 20% PSY 0.5%	${435.333\pm 12.014}^{\;b}$	${192.000\pm 2.000}^{\;a,b}$	${40.000\pm 2.000}^{\;b,c}$	${250.000\pm 2.000}^{\;b}$	${4.810\pm 0.295}^{\;c}$
WF 80%–QF 20% XG 0.5%	${394.000\pm 4.000}^{\;d}$	${198.333\pm 1.528}^{\;a}$	${24.333\pm 0.577}^{\;d}$	${224.000\pm 3.606}^{\;c}$	${8.153\pm 0.134}^{\;a}$
WF 80%–QF 20% PSY 0.25%/ XG 0.25%	${422.000\pm 7.550}^{\;b,c}$	${188.000\pm 1.000}^{\;b}$	${32.667\pm 2.082}^{\;c,d}$	${238.000\pm 5.568}^{\;b,c}$	${5.767\pm 0.350}^{\;b}$

P: dough tenacity; L: dough extensibility; W: baking strength; P/L: curve configuration ratio of tenacity and extensibility; ud: undetermined (high values detected). Dough samples contain WF: wheat flour, QF: quinoa flour, PSY: psyllium, and XG: xanthan gum. Values in the same column with different letters (a–e) are significantly different (One-way ANOVA with Tukey’s multiple comparison test, *p* < 0.05). Data are the mean ± standard deviation (n = 3).

**Table 4:** Physical analysis for bread loaf.

Bread Type/Parameter	*b/a*	$\mathrm{Specific}\;\mathrm{Volume}\;(\frac{{cm}^{3}}{g}$)	Weight Loss (%)	aw	pH
Wheat flour (WF)	1.320±0.000	${2.515\pm 0.007}^{\;a}$	${9.800\pm 0.000}^{\;b}$	${0.960\pm 0.000}^{\;a}$	${5.367\pm 0.035}^{\;a}$
WF 80%–QF 20%	1.460±0.000	${2.125\pm 0.007}^{\;d}$	${8.730\pm 0.000}^{\;d}$	${0.957\pm 0.012}^{\;a}$	${5.120\pm 0.000}^{\;b}$
WF 80%–QF 20% PSY 0.5%	1.320±0.000	${2.290\pm 0.000}^{\;c}$	${8.975\pm 0.064}^{\;c}$	${0.953\pm 0.006}^{\;a}$	${5.393\pm 0.021}^{\;a}$
WF 80%–QF 20% XG 0.5%	1.410±0.000	${2.295\pm 0.007}^{\;c}$	$10{.400\pm 0.000}^{\;a}$	${0.950\pm 0.000}^{\;a}$	${4.907\pm 0.012}^{\;c}$
WF 80%–QF 20% PSY 0.25/XG 0.25%	1.420±0.000	${2.350\pm 0.000}^{\;b}$	${7.940\pm 0.000}^{\;e}$	${0.960\pm 0.000}^{\;a}$	${5.147\pm 0.059}^{\;b}$

WF: wheat flour; QF: quinoa flour; PSY: psyllium; XG: xanthan gum; aw: water activity; characteristics of bread samples *b/a*: ratio width/height of slice. Kruskal–Wallis test was used to evaluate differences between treatments in terms of *b/a*. One-way ANOVA with Tukey’s multiple comparison tests, *p* < 0.05, evaluated significant differences between treatments for a specific volume, weight loss, aw, and pH [values in the same column with different letters (a–e) are significantly different]. Data are the mean ± standard deviation (n = 2).

**Table 5:** Physicochemical analyses for bread loaf and treatments.

9	Moisture (%)	Ash (%)	Lipids (%)	Protein (%)	Total Carbohydrates (%)	Crude Fiber (%)
Wheat flour (WF)	${32.930\pm 0.297}^{\;b}$	${0.800\pm 0.014}^{\;c}$	${9.875\pm 0.049}^{\;a}$	${7.565\pm 0.021}^{\;b}$	${48.830\pm 0.255}^{\;a}$	${0.610\pm 0.000}^{\;b}$
WF 80%–QF 20%	${32.590\pm 0.141}^{\;b,c}$	${1.035\pm 0.021}^{\;b}$	${9.895\pm 0.007}^{\;a}$	${9.085\pm 0.120}^{\;a}$	${47.395\pm 0.290}^{\;b}$	${0.525\pm 0.007}^{\;b}$
WF 80%–QF 20% PSY 0.5%	${34.115\pm 0.064}^{\;a}$	${1.760\pm 0.014}^{\;a}$	${9.150\pm 0.071}^{\;b}$	${8.900\pm 0.028}^{\;a}$	${46.075\pm 0.092}^{\;c}$	${0.745\pm 0.064}^{\;a}$
WF 80%-QF 20% XG 0.5%	${32.025\pm 0.007}^{\;c}$	${1.825\pm 0.021}^{\;a}$	${9.110\pm 0.085}^{\;b}$	${8.785\pm 0.021}^{\;a}$	${48.255\pm 0.049}^{\;a,b}$	${0.655\pm 0.021}^{\;a,b}$
WF 80%–QF 20% PSY 0.25/XG 0.25%	${32.535\pm 0.021}^{\;b,c}$	${1.805\pm 0.007}^{\;a}$	${9.185\pm 0.021}^{\;b}$	${8.625\pm 0.346}^{\;a}$	${47.850\pm 0.339}^{\;b}$	${0.760\pm 0.028}^{\;a}$

WF: wheat flour; QF: quinoa flour; PSY: psyllium; XG: xanthan gum. Values in the same column with different letters (a–c) are significantly different (One-way ANOVA with Tukey’s multiple comparison test, *p* < 0.05). Data are the mean ± standard deviation (n = 2).

**Table 6:** Crumb and crust color of bread loaf produced from wheat and quinoa composite flour.

Bread Type/Parameter	Crust Color			Crumb Color		
	L*	a*	b*	L*	a*	b*
Wheat flour (WF)	${52.890\pm 0.679}^{\;a}$	${15.083\pm 0.599}^{\;a}$	${36.383\pm 1.020}^{\;a}$	${64.710\pm 2.281}^{\;a}$	${-1.063\pm 0.101}^{\;c}$	${15.883\pm 0.624}^{\;c}$
WF 80%–QF 20%	${44.650\pm 0.665}^{\;c}$	${12.750\pm 0.563}^{\;b}$	${35.113\pm 0.350}^{\;a,b}$	${57.080\pm 0.389}^{\;b}$	${2.413\pm 0.405}^{\;b}$	${22.747\pm 0.540}^{\;b}$
WF 80%–QF 20% PSY 0.5%	${48.390\pm 1.787}^{\;b}$	${15.570\pm 0.632}^{\;a}$	${33.030\pm 2.511}^{\;a,b}$	${58.030\pm 1.630}^{\;b}$	${3.503\pm 0.350}^{\;a}$	${25.777\pm 0.955}^{\;a}$
WF 80%–QF 20% XG 0.5%	${46.333\pm 0.510}^{\;b,c}$	${16.113\pm 0.051}^{\;a}$	${33.040\pm 1.306}^{\;a,b}$	${59.037\pm 1.171}^{\;b}$	${2.500\pm 0.100}^{\;b}$	${23.910\pm 0.257}^{\;b}$
WF 80%–QF 20% PSY 0.25%/XG 0.25%	${48.230\pm 0.548}^{\;b}$	${15.837\pm 0.657}^{\;a}$	${31.797\pm 1.308}^{\;b}$	${63.323\pm 0.374}^{\;a}$	${2.293\pm 0.335}^{\;b}$	${23.943\pm 0.782}^{\;b}$

WF: wheat flour; QF: quinoa flour; PSY: psyllium; XG: xanthan gum. Values in the same column with different letters (a–c) are significantly different (One-way ANOVA with Tukey’s multiple comparison test, *p* < 0.05). Data are the mean ± standard deviation (n = 3).

## Data Availability

The original contributions presented in this study are included in the article. Further inquiries can be directed to the corresponding author.

## References

[1] United States Department of Agriculture (USDA) (2024). Foreign Agricultural Service.

[2] Rodríguez E., Lascano A., Sandoval G. (2012). Influence of the partial substitution of wheat flour for quinoa and potato flour on the thermomechanical and breadmaking properties of dough. Rev. UDCA Actual. Divulg. Científica.

[3] Ognean M., Jâşcanu V., Darie N., Popa L., Kurti A., Ognean C. (2007). Technological and nutritional and sensorial influences on using different types of hydrocolloids on bread. J. Agro Aliment. Process. Technol..

[4] Instituto Nacional de Estadística e Informática (INEI), Peru. https://proyectos.inei.gob.pe/microdatos/.

[5] Bewley J.D., Black M., Halmer P. (2006). The Encyclopedia of Seeds: Science, Technology and Uses.

[6] Rosentrater K.A., Evers A.D. (2018). Kent’s technology of cereals. An Introduction for Students of Food Science and Agriculture.

[7] Franco W., Evert K., Van Nieuwenhove C., Flour Q. (2021). Quinoa Flour, the Germinated grain Flour, and Sourdough as Alternative Sources for Gluten-Free Bread Formulation: Impact on Chemical, Textural and Sensorial Characteristics. Fermentation.

[8] (2024). World Health Organization. Obesity and Overweight; OMS. https://www.who.int/news-room/fact-sheets/detail/obesity-and-overweight.

[9] Coţovanu I., Mironeasa C., Mironeasa S. (2023). Nutritionally improved wheat bread supplemented with quinoa flour of large, medium and small particle sizes at typical doses. Plants.

[10] Ballester-Sánchez J., Yalcin E., Fernández-Espinar M.T., Haros C.M. (2019). Rheological and thermal properties of royal quinoa and wheat flour blends for breadmaking. Eur. Food Res. Technol..

[11] Wang X., Lao X., Bao Y., Guan X., Li C. (2021). Effect of whole quinoa flour substitution on the texture and in vitro starch digestibility of wheat bread. Food Hydrocoll..

[12] Mu J., Qi Y., Gong K., Chen Z., Brennan M., Ma Q., Wang J., Brennan C. (2023). Effects of quinoa flour (Chenopodium Quinoa Willd) substitution on wheat flour characteristics. Curr. Res. Food Sci..

[13] Coțovanu I., Ungureanu-Iuga M., Mironeasa S. (2021). Investigation of quinoa seeds fractions and their application in wheat bread production. Plants.

[14] El-Sohaimy S.A., Shehata M.G., Mehany T., Zeitoun M.A. (2019). Nutritional, physicochemical, and sensorial evaluation of flat bread supplemented with quinoa flour. Int. J. Food Sci..

[15] Rodriguez-Sandoval E., Sandoval G., Cortes-Rodríguez M. (2012). Effect of quinoa and potato flours on the thermomechanical and breadmaking properties of wheat flour. Braz. J. Chem. Eng..

[16] Moawad E., Rizk I., Kishk Y., Youssif M. (2018). Effect of substitution of wheat flour with quinoa flour on quality of pan bread and biscuit. Arab. Univ. J. Agric. Sci. (AUJAS).

[17] Man S.M., Păucean A., Muste S., Pop A., Muresan E.A. (2017). Influence of Psyllium husk (Plantago ovata) on bread quality. BUASVMCN-FST.

[18] Filipčev B., Pojić M., Šimurina O., Mišan A., Mandić A. (2021). Psyllium as an improver in gluten-Free breads: Effect on volume, crumb texture, moisture binding and staling kinetics. LWT.

[19] Santos F.G., Capriles V.D. (2021). Relationships between dough thermomechanical parameters and physical and sensory properties of gluten-Free bread texture during storage. LWT.

[20] Aboulnaga E.A., Ibrahim F.Y., Youssif M.R.G., Mohamed A.M.I. (2018). Influence of various hydrocolloids addition on pan bread quality. J. Food Dairy. Sci..

[21] Belorio M., Marcondes G., Gómez M. (2020). Influence of Psyllium versus xanthan gum in starch Properties. Food Hydrocoll..

[22] Instituto Nacional de Calidad (2016). Norma Técnica Peruana. Determinación de la Humedad.

[23] Instituto Nacional de Calidad (2016). Norma Técnica Peruana. Harinas. Determinación de Ceniza.

[24] AACC International (2000). Method 76-33.01. damaged starch—Amperometric method by SDmatic. Approved Methods of the American Association of Cereal Chemists.

[25] AACC International (2000). Method 56-81.04. determination of falling number. Approved Methods of the American Association of Cereal Chemists.

[26] Giese J. (1995). Measuring physical properties of foods. Food Technol..

[27] AOAC (2012). Association of Official Analytical Chemists. Official Method of Analysis.

[28] ICC (1992). Method for Using the Chopin Alveograph. ICC Standard Method No. 121. Standard Methods of the International Association for Cereal Science and Technology.

[29] AACC (2000). Approved Methods of the American Association of Cereal Chemists.

[30] Aguirre E., Rodriguez G., León Lopez A., Urbina-Castillo K., Villanueva E. (2021). Incorporation of chia seeds (*Salvia hispanica* L.) in cereal flour mixtures: Rheology and quality of sliced bread. Dyna.

[31] Tamba-Berehoiu R.-M., Turtoi O.M., Popa N.C. (2019). Assessment of quinoa flours effect on wheat flour doughs rheology and bread quality. Ann. UDJG Food Technol..

[32] American Association of Cereal Chemists (2000). Method 10-05.01. guideliness for measurement of volumen by rapeseed displacement. Approved Methods of Analysis.

[33] Horstmann S.W., Belz M.C.E., Heitmann M., Zannini E., Arendt E.K. (2016). Fundamental study on the impact of gluten-Free starches on the quality of gluten-Free model breads. Foods.

[34] Machado Alencar N.M., Steel C.J., Alvim I.D., de Morais E.C., Andre Bolini H.M. (2015). Addition of quinoa and amaranth flour in gluten-Free breads: Temporal profile and instrumental analysis. LWT Food Sci. Technol..

[35] Encina-Zelada C.R., Cadavez V., Monteiro F., Teixeira J.A., Gonzales-Barron U. (2018). Combined effect of xanthan gum and water content on physicochemical and textural properties of gluten-Free batter and bread. Food Res. Int..

[36] Instituto Nacional de Calidad (2011). Norma Técnica Peruana. NTP 205.003:1980. (Revisada el 2011).

[37] Reyes M., Gómez I., Espinoza C. (2017). Tablas Peruanas de Composición de Alimentos.

[38] Instituto Nacional de Calidad (2015). Norma Técnica Peruana. NTP 205.064:2015. Trigo. Harina de Trigo para Consumo Humano. Requisitos.

[39] Best I., Portugal A., Casimiro-Gonzales S., Aguilar L., Ramos-Escudero F., Honorio Z., Rojas-Villa N., Benavente C., Muñoz A.M. (2023). Physicochemical and Rheological Characteristics of Commercial and Monovarietal Wheat Flours from Peru. Foods.

[40] Franco M., Gómez M. (2022). Effect of Psyllium on physical properties, composition and acceptability of whole grain breads. Foods.

[41] El-Said E.T., Soliman A.S., Abbas M.S., Aly S.E. (2021). Treatment of anaemia and malnutrition by Shamy bread fortified with Spirulina, quinoa and chickpea flour. Egypt. J. Chem..

[42] Urbina Dicao K.S., Santacruz Terán S.G., Guapi Álava G.M., Revilla Escobar K., Aldas Morejon J.P. (2023). Physicochemical characterization of cereals grains and functionality of amaranth (*Amaranthus caudatus*) and quinoa (*Chenopodium quinoa*) flours. Rev. Colomb. Investig. Agroindustriales.

[43] Instituto Nacional de Calidad (2019). Norma Técnica Peruana. NTP 011.451:2018. Granos Andinos. Harina de Quinua. Requisitos.

[44] Li M., Peng J., Zhu K.-X., Guo X.-N., Zhang M., Peng W., Zhou H.-M. (2013). Delineating the microbial and physical–chemical changes during storage of ozone treated wheat flour. Innov. Food Sci. Emerg. Technol..

[45] Gautam R.K., Goswami M., Mishra R.K., Chaturvedi P., Awashthi M.K., Singh R.S., Giri B.S., Pandey A. (2021). Biochar for remediation of agrochemicals and synthetic organic dyes from environmental samples: A review. Chemosphere.

[46] Awoyale W., Asiedu R., Kawalawu W.K.C., Abass A., Maziya-Dixon B., Kromah A., Edet M., Mulbah S. (2020). Assessment of the suitability of different cassava varieties for Gari and fufu flour production in Liberia. Asian Food Sci. J..

[47] Katyal M., Virdi A.S., Kaur A., Singh N., Kaur S., Ahlawat A.K., Singh A.M. (2016). Diversity in Quality Traits amongst Indian Wheat Varieties I: Flour and protein characteristics. Food Chem..

[48] Kundu M., Khatkar B.S., Gulia N. (2017). Assessment of chapatti quality of wheat varieties based on physicochemical, rheological and sensory traits. Food Chem..

[49] Rababah T., Alu’datt M., Al-Mahasneh M., Gammoh S., Al-Obaidy M., Ajouly T., Bartkute-Norkūniene V. (2019). The effect of different fl our extraction rates on physiochemical and rheological characteristics. Bulg. J. Agric. Sci..

[50] Jaldani S.H., Nasehi B., Barzegar H., Sepahvand N. (2017). The effects of adding quinoa flour and xanthan gum on the chemical and sensory properties of Barbari bread using response surface methodology. Iran. J. Food Sci. Technol..

[51] Barak S., Mudgil D., Khatkar B.S. (2014). Effect of flour particle size and damaged starch on the quality of cookies. J. Food Sci. Technol..

[52] Solaesa Á.G., Villanueva M., Vela A.J., Ronda F. (2020). Protein and lipid enrichment of quinoa (cv. Titicaca) by dry fractionation. Techno-functional, thermal and rheological properties of milling fractions. Food Hydrocoll..

[53] Jukić M., Komlenić D., Mastanjević K.M., Mastanjević K., Lučan M., Popovici C., Nakov G., Lukinac J. (2019). Influence of damaged starch on the quality parameters of wheat dough and bread. Ukr. Food J..

[54] Srichuwong S., Curti D., Austin S., King R., Lamothe L., Gloria-Hernandez H. (2017). Physicochemical properties and starch digestibility of whole grain sorghums, millet, quinoa and amaranth flours, as affected by starch and non-starch constituents. Food Chem..

[55] CHOPIN (2010). Alveógrafo NG Consistografo: Modo de Empleo. https://es.scribd.com/document/413339528/Manual-40-Es-Alveograph-Ng.

[56] Dussan-Sarria S., Hurtado-Hurtado D.L., Camacho-Tamayo J.H., Granulometría P. (2019). Granulometría, Propiedades Funcionales y Propiedades de Color de las Harinas de Quinua y Chontaduro. Inf. Tecnol..

[57] Rojas-Garbanzo C., Pérez A.M., Vaillant F., Pineda-Castro M.L. (2016). Physicochemical and antioxidant composition of fresh peach palm (*Bactris gasipaes* Kunth) fruits in Costa Rica. Braz. J. Food Technol..

[58] Delatte S., Doran L., Blecker C., De Mol G.D., Roiseux O., Gofflot S., Malumba P. (2019). Effect of pilot-scale steam treatment and endogenous alpha-amylase activity on wheat flour functional properties. J. Cereal Sci..

[59] Mironeasa S., Codină G.G., Popa C. (2013). Effect of the addition of Psyllium fiber on wheat flour dough rheological properties. Recent. Res. Med. Biol. Biosci..

[60] Mellado Z.M. (2003). Falling number un análisis para evaluar la calidad de la harina. Tierra Adentro.

[61] Kiszonas A.M., Engle D.A., Pierantoni L.A., Morris C.F. (2018). Relationships between Falling Number, α-amylase activity, milling, cookie, and sponge cake quality of soft white wheat. Cereal Chem..

[62] Joshi A.A., Kshirsagar R.B., Sadawarte S.K., Patil B.M., Sawate A.R. (2022). Comparative evaluation of baking and functional qualities of black wheat flour. Pharm. Innov. J..

[63] Gallagher E., Gormley T.R., Arendt E.K. (2003). Crust and crumb characteristics of gluten Free breads. J. Food Eng..

[64] Jødal A.S.S., Larsen K.L. (2021). Investigation of the relationships between the alveograph parameters. Sci. Rep..

[65] Edwars N., Dexter J. (1987). Alveograph—Sources of problems in curve interpretation with hard common wheat flour. Can. Inst. Food Sci. Technol..

[66] Coțovanu I., Mironeasa S. (2021). Buckwheat seeds: Impact of milling fractions and addition level on wheat bread dough rheology. Appl. Sci..

[67] Cappelli A., Cini E., Guerrini L., Masella P., Angeloni G., Parenti A. (2018). Predictive models of the rheological properties and optimal water content in doughs: An application to ancient grain flours with different degrees of refining. J. Cereal Sci..

[68] US Department of Agriculture (USDA) Agriculture Research Service. Xanthan Gum 2019. https://fdc.nal.usda.gov/fdc-app.html#/food-details/452087/nutrients.

[69] Rosell C.M., Santos E., Collar C. (2010). Physical characterization of fiber-enriched bread doughs by dual mixing and temperature constraint using the Mixolab^®^. Eur. Food Res. Technol..

[70] Khalid K.H., Ohm J.-B., Simsek S. (2017). Whole wheat bread: Effect of bran fractions on dough and end-product quality. J. Cereal Sci..

[71] Aldughpassi A., Zafar T., Sidhu J.S., Al-Hassawi F., Abdullah M.M., Al-Othman A. (2020). Effect of Psyllium husk, bran, and raw wheat germ addition on the rheological characteristics of Arabic (pita) bread dough. Int. J. Food Sci..

[72] Tebben L., Li Y. (2019). Effect of xanthan gum on dough properties and bread qualities made from whole wheat flour. Cereal Chem..

[73] Barber B., Ortolá C., Barber S., Fernández F. (1992). Storage of packaged white bread. Z. Lebensm. Unters. Forch..

[74] Fratelli C., Santos F.G., Muniz D.G., Habu S., Braga A.R.C., Capriles V.D. (2021). Psyllium Improves the Quality and Shelf Life of Gluten-Free Bread. Foods.

[75] Sharoba A.M.A., El-Desouky A.I., Mahmoud M.H.M., Youssef K.M. (2009). Quality attributes of some breads made from wheat flour substituted by different levels of whole amaranth meal. J. Food Dairy. Sci..

[76] Shittu T.A., Aminu R.A., Abulude E.O. (2009). Functional effects of xanthan gum on composite cassava-wheat dough and bread. Food Hydrocoll..

[77] Zannini E., Waters D.M., Arendt E.K. (2014). The Application of dextran compared to other hydrocolloids as a novel food ingredient to compensate for low protein in biscuit and wholemeal wheat flour. Eur. Food Res. Technol..

[78] Wang S., Opassathavorn A., Zhu F. (2015). Influence of quinoa flour on quality characteristics of cookie, bread and Chinese steamed bread. J. Texture Stud..

[79] Tsanasidou C., Kosma I., Badeka A., Kontominas M. (2021). Quality parameters of wheat bread with the addition of untreated cheese whey. Molecules.

[80] Maneffa A.J., Stenner R., Matharu A.S., Clark J.H., Matubayasi N., Shimizu S. (2017). Water activity in liquid food systems: A molecular scale interpretation. Food Chem..

[81] Rosell C.M., Rojas J.A., Benedito de Barber C. (2001). Influence of hydrocolloids on dough rheology and bread quality. Food Hydrocoll..

[82] Salazar D., Naranjo M., Perez L., Valencia A., Acurio L., Gallegos L., Alvarez F., Amacha P., Valencia M., Rodriguez C. (2017). Development of a new bread enrichment with quinoa flour and whey. IOP Conf. Ser. Earth Environ. Sci..

[83] Haros C.M., Reguera M., Sammán N., Paredes-López O. (2023). Latin American Seeds. Agronomic Processing and Health Aspects.

[84] Rosell C.M., Cortez G., Repo-Carrasco R. (2009). Breadmaking use of Andean crops quinoa, kañiwa, Kiwicha, and tarwi. Cereal Chem..

[85] Chisenga S.M., Workneh T.S., Bultosa G., Alimi B.A., Siwela M. (2020). Dough rheology and loaf quality of wheat-cassava bread using different cassava varieties and wheat substitution levels. Food Biosci..

